# Distribution of Tobacco and Nicotine Use Indicators from the Periodic Health Assessment and Medical Diagnostic Codes Among U.S. Active Component Service Members, 2023

**Published:** 2026-05-15

**Authors:** Kristen R. Rossi, Scott J. Russell, Sithembile L. Mabila

**Affiliations:** Epidemiology and Analysis Branch, Armed Forces Health Surveillance Division, Public Health Directorate, Defense Health Agency, Silver Spring, MD: Ms. Rossi, Mr. Russell, Dr. Mabila

## Abstract

Military service members remain a priority population for assessing the prevalence, patterns, and long-term consequences of tobacco and nicotine use. The limitations inherent to documenting use among military service members, however, complicate the design of exposure assessment. This study combined 2 data sources—by aggregating self-reported Periodic Health Assessment (PHA) survey data with International Classification of Diseases, 9th and 10th revisions, Clinical Modification (ICD-9-CM / ICD-10-CM) medical diagnostic codes—to classify nicotine and tobacco use as exposures delineated by recent use or history of any use. The study population included a total of 921,394 U.S. active component service members who completed a PHA in 2023. PHA classification for ‘recent use’ was defined by self-reported use of any tobacco or nicotine product within the past 30 days, whereas ‘history of any use’ included recent users in addition to those who reported cessation of use. The full roster of service members who completed the PHA in 2023 was matched to ambulatory and inpatient medical records within 30 days, before or after, the PHA sample period (December 1, 2022–January 31, 2024) to identify selected ICD-10-CM codes for recent use. Selected diagnostic codes for a ‘history of any use’ were queried for a period of 20 years preceding and 30 days following (January 1, 2004–January 31, 2024) the PHA sample period. Among PHA respondents, 22.0% (n=203,156) self-reported recent nicotine or tobacco use. When aggregating PHA data with recent exposure classified from diagnostic codes, the resulting assessment of recent nicotine or tobacco use increased to 28.7% (n=264,194). Critically, this aggregation identified 61,038 U.S. service members with no evidence of recent use on the PHA but with a concurrent clinical record during the specified matching period. Aggregating data sources for a history of any use only nominally improved the estimate, increasing it from 41.1% (PHA alone) to 43.1%. Agreement between sources was fair for both recent use (κ=0.28) and historical use (κ=0.36). The results of this study indicate that neither self-reported PHA data nor medical diagnostic codes alone provide a complete picture of tobacco and nicotine use among U.S. active component service members.

**What are the new findings?**

The combination of medical diagnostic codes with self-reported PHA survey responses increases exposure estimates of recent tobacco or nicotine use among U.S. active component service members to 28.7%, in comparison to 22.0% if exclusively assessing recent use from the PHA.

**What is the impact on readiness and force health protection?**

The integration of multiple data sources may provide a more comprehensive assessment of recent nicotine and tobacco exposure among service members, directly supporting enhanced public health surveillance.

A 2014 Surgeon General's report highlighted tobacco and nicotine use in the U.S. Armed Forces as a focus of careful study, underscoring a critical need for robust and continuous public health surveillance methods. ^1^ This need persists today, as the U.S. Surgeon General has identified U.S. military service members as a priority population for developing effective prevention and cessation programs. ^2^ The prevalence of tobacco use remains higher among active duty service members compared to the general U.S. population, compounding the issue of reduced military readiness. ^3^ The effects on short-term health and operational effectiveness are significant, impairing physical endurance, cognitive function and vision, while also slowing recovery from injury. ^4^ Furthermore, smoking degrades readiness by increasing the risk of work absenteeism, respiratory infections, and complications with wound healing. ^4^

In the U.S. Department of War (DOW), several data sources are available to assess service members' tobacco and nicotine use, each characterized by different strengths and limitations. The Health Related Behaviors Survey (HRBS), a DOW flagship survey, in 2018 estimated 37.8% of service members currently used tobacco in some form, such as combustible cigarettes, e-cigarettes, cigars, smokeless tobacco, pipes, or hookahs. Limitations from the latest HRBS publication note that the response rate (9.6%) is considered low for survey research, which can result in a non-representative sample and affect the accuracy of prevalence estimates. ^5^ The DOW's annual Periodic Health Assessment (PHA) offers a more representative sample of service members, as a mandatory annual requirement to assess currency of individual medical readiness. ^6^ While survey data from the PHA provide specific, individual exposures by asking about a range of tobacco and nicotine use within the last 30 days, length of use, and past use, the mandated nature of the assessment may lead to under-reporting when compared to the confidential HRBS. ^7^

Diagnostic codes from medical records, such as the International Classification of Diseases, 9th and 10th revisions, Clinical Modification (ICD-9-CM / ICD-10-CM) codes, provide an alternate measure. These include codes for current nicotine dependence, tobacco use, and a personal history of nicotine dependence. Use of these diagnostic codes have been shown to substantially under-estimate the prevalence of use, however, when compared to survey data. ^8 , 9^ This under-estimation may be influenced by service-branch-specific cultural norms and attitudes towards tobacco use, as well as a potential reluctance among service members to disclose behaviors to health care providers that could be perceived negatively or affect their careers. ^10 , 11^

These inherent limitations associated with the documentation of tobacco and nicotine use complicate the design of epidemiological studies, including those measuring prevalence and incidence, monitoring trends, and assessing risk factors for various health outcomes. Furthermore, the longitudinal characterization of use presents methodological challenges, as individuals may initiate, quit, and relapse multiple times throughout their military careers. The primary objective of this study was to evaluate the concordance between 2 distinct data sources—self-reported surveys from the Periodic Health Assessment (PHA) and administrative medical diagnostic codes—to classify all-inclusive categories for tobacco and nicotine use. This study specifically measured and compared the level of agreement of classifications for “recent nicotine or tobacco use” and “history of any nicotine or tobacco use,” to understand the unique contribution of each data source. By characterizing the distribution of data for this complex behavior, these results are intended to inform the development of standardized classifications by highlighting the strengths and limitations of relying upon any single data source alone.

## Methods

This analysis included active component service members (ACSMs) of the U.S. Army, Navy, Air Force, Marine Corps, and Coast Guard; Space Force ACSMs were included with the Air Force. A roster of service members who completed a PHA during 2023, regardless of any tobacco or nicotine use responses, was compiled. Per DOW Instruction 6200.06, service members are required to complete a PHA every 12 months. The PHA provides an annual, standardized health assessment for U.S. military service members to assess individual medical readiness, including occupational and environmental health evaluations, with provision of evidence-based preventive health information and recommendations. ^6^

The full roster of service members who completed PHA during 2023, regardless of any tobacco or nicotine use responses, was matched by unique identifiers to query ambulatory and inpatient medical records for each ACSM maintained in the Defense Medical Surveillance System (DMSS). DMSS contains all encounters in military medical and civilian treatment facilities when reimbursed through the Military Health System. From the PHA responses and applicable medical records, evidence of exposure to tobacco or nicotine was classified into 2 separate outcomes: 1) recent use or 2) history of any use.

### Recent use

As shown in **Table 1** , a service member was classified as a ‘recent user’ from the PHA if that service member reported use of any listed tobacco or nicotine product (e.g., cigarette, e-cigarette, chewing tobacco) on at least 1 occasion within the past 30 days. To identify recent tobacco or nicotine use from medical diagnostic codes, we examined medical records for relevant diagnostic codes documented within 30 days, before or after, the PHA sample period (December 1, 2022–January 31, 2024). This method established a standardized documentation period corresponding to the 30-day use period assessed in the survey. Inpatient and ambulatory care records with ICD-10-CM diagnostic codes for nicotine dependence (F17.2*), tobacco use (Z72.0), and tobacco use disorder complicating pregnancy, childbirth and puerperium (O99.33*) were queried from any diagnostic position to document recent nicotine or tobacco use. For the purposes of this study, nicotine dependence and tobacco use disorders were broadly classified as recent tobacco and nicotine use. Service members with resulting medical records matched for these diagnostic codes were classified as positive for diagnostic classification of recent nicotine or tobacco use; conversely, if no applicable medical record was matched, the diagnostic classification was coded as negative.

**TABLE 1.** Classifications for Tobacco and Nicotine Recent Use, History of Any Use from the Periodic Health Assessment and Medical Diagnostic Codes

Periodic Health Assessment		
Definition	PHA Survey Item	Response
Recent use	In the past 30 days, which of the following products have you used on at least 1 day?	Used once in the last 30 days: cigarettes
		Used once in last 30 days: cigars
		Used once in last 30 days: chewing tobacco
		Used once in last 30 days: electronic cigarettes
		Used once in last 30 days: hookahs
		Used once in last 30 days: pipes
		Used once in last 30 days: snus
		Used once in last 30 days: dissolvable tobacco
		Used once in last 30 days: bidis
		Used once in last 30 days: other tobacco product
For history of any use, include recent use survey items, plus:	Which of the following describes your past tobacco use?	I used tobacco in the past, but quit.
ICD-9-CM and ICD-10-CM Diagnostic Codes ^a^		
Definition	Codes	Description
Recent use	ICD-10-CM: F17.2*; ICD-9-CM: 305.1	Nicotine dependence; tobacco use disorder
	ICD-10-CM: Z72.0	Tobacco use
	ICD-10-CM: O99.33*; ICD-9-CM: 649.0*	Tobacco use disorder complicating pregnancy, childbirth, and the puerperium
For history of any use, include recent use codes, plus:	ICD-10-CM: Z87.891; ICD-9-CM: V15.82	Personal history of nicotine dependence ^b^

Abbreviations: ICD-9-CM, International Classification of Diseases, 9th Revision; ICD-10-CM, International Classification of Diseases, 10th Revision.

a ICD-10-CM code for ‘tobacco use’ was introduced in 2015; there is no comparable ICD-9-CM code. Prior to 2015, the only code referencing ‘tobacco use’ was ICD-9-CM 305.1 (‘tobacco use disorder’), which was used to indicate diagnosis of tobacco dependence. For the purposes of this study, nicotine dependence, tobacco use disorders, or history of nicotine dependence are broadly classified as past history tobacco and nicotine use.

b This code denotes personal history of nicotine dependence, signifying that the individual previously experienced nicotine dependence but was not currently experiencing it.

### History of any use

The classification parameters for documenting a history of any tobacco or nicotine use are shown in **Table 1** ; this broader method aims to identify both recent and former users. The PHA classification includes everyone identified by the ‘recent use’ method, but it expands to capture individuals who select the response “I used tobacco in the past, but quit.” To define a history of any nicotine or tobacco use from medical diagnostic codes, applicable medical records were queried for the preceding 20 years through 30 days following the PHA completion period (January 1, 2004–January 31, 2024). Inpatient and ambulatory care records with ICD-9-CM/ICD-10-CM diagnostic codes for nicotine dependence (305.1 / F17.2*), tobacco use (Z72.0), tobacco use disorder complicating pregnancy, childbirth and puerperium (649.0* / O99.33*), and personal history of nicotine dependence (Z87.891 / V15.82) were queried from any diagnostic position. An ICD-10-CM code for ‘tobacco use’ was introduced in 2015; there is no comparable ICD-9-CM code. Prior to 2015, the only ICD code referencing ‘tobacco use’ was ICD-9-CM 305.1 (‘tobacco use disorder’), which was used to indicate a diagnosis of tobacco dependence. For the purposes of this study, nicotine dependence, tobacco use disorders, or a history of nicotine dependence were broadly classified as past history of tobacco and nicotine use. Service members with resulting medical records matched for these diagnostic codes were classified as positive for diagnostic classification of any nicotine or tobacco use; conversely, if no applicable medical record was matched, diagnostic classification was coded as negative.

### Analysis

The dichotomous outcomes for tobacco and nicotine ‘recent use’ and ‘history of any use’ were stratified to assess overlap in classification exposure by data source. Cohen's kappa was calculated to examine level of agreement between self-reported PHA responses and diagnostic code documentation. To examine demo-graphic differences of recent or history of tobacco or nicotine use by data source classification, we investigated the differences between branch of service, sex, categorized age at date of completion, and racial and ethnic group by separating responses into 1 of the 3 data source categories: concurrent PHA and diagnostic code documentation, exclusive PHA documentation, and exclusive diagnostic code documentation.

## Results

### Recent use

A total of 921,394 U.S. ACSMs completed a PHA documented in 2023 **(Table 2)** . Among those service members, 22.0% (n=203,156) self-reported recent nicotine or tobacco use on the PHA. The most frequent responses for tobacco or nicotine use within the last 30 days were reported for electronic cigarettes (n=115,486), cigarettes (n=47,325), chewing tobacco (n=45,777), cigars (n=21,517), and other tobacco products (n=9,630).

**TABLE 2.** Exposure Classification Agreement for Tobacco or Nicotine Use by the Periodic Health Assessment and Medical Diagnostic Codes ^a^

Recent Nicotine or Tobacco Use					
		PHA (+)	PHA (‒)	Row Totals	
		No.	No.	No.	%
Diagnostic code (+)	*n*	65,739	61,038	126,777	13.8
Diagnostic code (‒)	*n*	137,417	657,200	794,617	86.2
Column totals	*n*	203,156	718,238	921,394	100
	%	22.0	78.0	κ: 0.2758 (0.2734, 0.2781)	
History of Any Nicotine or Tobacco Use					
		PHA (+)	PHA (‒)	Row Totals	
		No.	No.	No.	%
Diagnostic code (+)	*n*	137,365	18,787	156,152	16.9
Diagnostic code (‒)	*n*	241,298	523,944	765,242	83.1
Column totals	*n*	378,663	542,731	921,394	100
	%	41.1	58.9	κ: 0.3601 (0.3584, 0.3619)	

Abbreviations: +, positive response; ‒, negative response; No., number; *n* , number; PHA, Periodic Health Assessment; κ, Cohen's kappa.

a Based on sample of active component service members who completed a PHA in 2023.

Among the 921,394 ACSMs who completed a PHA in 2023, 126,777 (13.8%) had a medical record with a diagnostic code for nicotine dependence (n=66,528) or tobacco use (n=88,519) during the period December 1, 2022–January 31, 2024; few service members had a diagnostic code for tobacco use disorder complicating pregnancy (n=1,212) (data not shown). Aggregation of PHA data with exposure determination captured exclusively from diagnostic codes (n=61,038) increased the estimate of recent nicotine or tobacco use to 28.7% (n=264,194). If exposure assessment was limited to medical records, independent of PHA responses, the estimated recent use of tobacco or nicotine exposure for this sample reduced to 13.8% (n=126,777). The Cohen's kappa statistic of 0.28 indicates a fair level of agreement between the PHA and diagnostic codes for identifying recent tobacco or nicotine use **(Table 2)** .

The demographic distribution of the 264,194 recent tobacco or nicotine users was examined for ACSMs with exclusive PHA exposure (n=137,417, 52.0%), diagnostic coding exclusivity (n=61,038, 23.1%), and concurrent data sources (n=65,739, 24.9%) **(Table 2)** . Compared to other services, the Marine Corps was over-represented in exclusive PHA data documentation versus exclusive diagnostic data (66.1% vs 15.2%). Documentation of tobacco or nicotine use exclusively from the PHA decreased with age, with a substantial difference observed for ACSMs younger than age 25 years (82.0% from PHA vs. 6.7% from diagnostic codes) **(Table 3)** .

**TABLE 3.** Estimates of Recent Tobacco or Nicotine Use, Classified by Self-Reporting from the Periodic Health Assessment or Medical Diagnostic Codes ^a^

	Concurrent PHA and Diagnostic Code Documentation		Exclusive Self-Reporting from PHA		Exclusive Documentation from Diagnostic Codes		All
	No.	%	No.	%	No.	%	No.
Total	65,739	24.9	137,417	52.0	61,038	23.1	264,194
Branch of service							
Army	31,468	27.3	57,059	49.6	26,574	23.1	115,101
Navy	12,119	25.8	24,200	51.6	10,576	22.6	46,895
Air Force ^b^	14,215	23.3	30,099	49.2	16,805	27.5	61,119
Marine Corps	6,489	18.7	22,984	66.1	5,287	15.2	34,760
Coast Guard	1,447	22.9	3,073	48.7	1,792	28.4	6,312
Unknown ^c^	1	14.3	2	28.6	4	57.1	7
Sex							
Male	59,283	25.3	123,017	52.4	52,282	22.3	234,582
Female	6,456	21.8	14,400	48.6	8,756	29.6	29,612
Age, *y*							
Unknown	0	0.0	1	100	0	0.0	1
<25	8,605	11.4	61,944	82.0	5,027	6.7	75,576
25–29	14,445	21.8	41,373	62.5	10,342	15.6	66,160
30–34	15,660	31.6	18,735	37.8	15,118	30.5	49,513
35–39	15,851	37.0	9,755	22.8	17,179	40.2	42,785
40+	11,178	37.1	5,609	18.6	13,372	44.3	30,159
Race and ethnicity							
White, non-Hispanic	41,253	26.5	77,437	49.8	36,927	23.7	155,617
Black, non-Hispanic	8,209	23.3	19,505	55.3	7,528	21.4	35,242
Hispanic	8,050	19.3	24,413	58.4	9,351	22.4	41,814
Other	8,227	26.1	16,062	51.0	7,232	22.9	31,521
Military rank							
Enlisted	59,377	25.2	125,660	53.4	50,182	21.3	235,219
Officer	6,360	22.0	11,719	40.5	10,851	37.5	28,930
Unknown	2	4.4	38	84.4	5	11.1	45

Abbreviations: PHA, Periodic Health Assessment; No., number; *y* , years.

a Based on sample of active component service members who completed a PHA in 2023.

b Includes Space Force members.

c Service members documented as active duty but branch of service unknown.

### History of any use

Of the 921,394 ACSMs who completed a PHA in 2023, 41.1% (n=378,663) self-reported a history of any nicotine or tobacco use **(Table 2)** . A total of 176,004 ACSMs reported using tobacco in the past but had currently quit, whereas all other ACSMs were classified as ‘any use’ from the ‘recent use’ PHA classification criterion.

After matching the PHA respondent roster to medical records for the period January 1, 2004–January 31, 2024, a total of 156,152 ACSMs had an applicable medical record with evidence of any history of tobacco or nicotine use. A total of 52,370 ACSMs had a record for a personal history of nicotine dependence, which did not exceed the total number of medical records identified with diagnoses for tobacco use (n=93,919) or nicotine dependence (n=71,219) (data not shown). Aggregating PHA data with exposure determination captured exclusively from diagnostic codes (n=18,787) only increased the history of any use estimate to 43.1% (n=397,450). If a historical exposure assessment for tobacco or nicotine use was limited to medical records, independent of PHA responses, the estimated ‘any use’ of tobacco or nicotine exposure for this sample drops to 16.9% (n=156,152) The Cohen's kappa statistic of 0.36 indicates a fair level of agreement between the PHA and diagnostic codes for identifying a history of any tobacco or nicotine use **(Table 2)** .

The demographic distribution of the 397,450 ACSMs with a history of tobacco or nicotine use was examined for those with exclusive PHA exposure (n=241,298, 60.7%), diagnostic coding exclusivity (n=18,787, 4.7%), and concurrent data sources (n=137,365, 34.6%) **(Table 4)** . The Marine Corps represented the highest service-specific proportion of exclusive PHA documentation for tobacco or nicotine use (71.8%) and, consequentially, low-est concurrent PHA and diagnostic code documentation (24.8%). Exclusive PHA documentation decreased with age, while concurrent PHA and diagnostic code documentation increased with age.

**TABLE 4.** Estimates of Any History of Tobacco or Nicotine Use, Classified by Self-Reporting from the Periodic Health Assessment or Diagnostic Codes ^a^

	Concurrent PHA and Diagnostic Code Documentation		Exclusive Self-Reporting from PHA		Exclusive Documentation from Diagnostic Codes		All
	No.	%	No.	%	No.	%	No.
Total	137,365	34.6	241,298	60.7	18,787	4.7	397,450
Branch of service							
Army	60,154	36.8	94,980	58.0	8,527	5.2	163,661
Navy	25,377	34.8	44,366	60.9	3,082	4.2	72,825
Air Force ^b^	35,367	35.4	59,445	59.5	5,020	5.0	99,832
Marine Corps	12,429	24.8	35,995	71.8	1,705	3.4	50,129
Coast Guard	4,033	36.7	6,510	59.2	452	4.1	10,995
Unknown ^c^	5	62.5	2	25.0	1	12.5	8
Sex							
Male	120,492	34.4	215,231	61.4	14,910	4.3	350,633
Female	16,873	36.0	26,067	55.7	3,877	8.3	46,817
Age, *y*							
Unknown	13,654	12.9	89,179	84.2	3,097	2.9	105,930
<25	26,477	25.7	72,772	70.6	3,857	3.7	103,106
25–29	32,503	42.7	39,402	51.7	4,252	5.6	76,157
30–34	36,199	57.2	22,865	36.2	4,185	6.6	63,249
35–39	28,531	58.2	17,080	34.9	3,396	6.9	49,007
40+	1	100	—	0.0	—	0.0	1
Race and ethnicity							
White, non-Hispanic	86,176	37.0	137,490	59.0	9,466	4.1	233,132
Black, non-Hispanic	15,658	31.6	30,333	61.2	3,602	7.3	49,593
Hispanic	18,531	27.7	44,696	66.9	3,574	5.4	66,801
Other	17,000	35.5	28,779	60.1	2,145	4.5	47,924
Military rank							
Enlisted	118,855	34.8	208,060	60.9	14,964	4.4	341,879
Officer	18,502	33.3	33,161	59.8	3,823	6.9	55,486
Unknown	8	9.4	77	90.6	—	0.0	85

Abbreviations: PHA. Periodic Health Assessment; No., number; *y* , years.

a Based on sample of active component service members who completed a PHA in 2023.

b Includes Space Force members.

c Service members documented as active duty but branch of service unknown.

## Discussion

The results of this study indicate that neither self-reported PHA data nor medical diagnostic codes alone provide a complete picture for all-inclusive classifications of tobacco and nicotine use among ACSMs. To identify recent use, reliance on either PHA data or diagnostic codes in isolation leads to significant under-estimation. Aggregating the 2 sources increased the captured population from 22.0% (PHA alone) to 28.7%. Analysis revealed that over 61,000 recent users would be missed without the inclusion of diagnostic codes. Conversely, the addition of 2 decades of medical records only marginally increased an estimate for history of any use from 41.1% (PHA alone) to 43.1%.

These findings contextualize existing data sources for all-inclusive classifications of tobacco and nicotine use, ranging from self-reported behaviors, clinically documented use, and diagnosed disorders. By comparing self-reported data from the PHA against administrative medical diagnostic codes, this study sought to understand the unique contribution of each source. The Armed Forces Health Surveillance Division (AFHSD) currently maintains standardized classifications for covariates that may typically be included in investigations, such as clinical overweight/obesity and non-medical factors influencing health (e.g., social, environmental and behavioral factors) ^12 , 13^ ; however, tobacco and nicotine use, in addition to other substance abuse disorders such as alcohol dependence, are not currently represented in a standardized surveillance case definition form. The results from this study are intended to inform the development of standardized classifications by highlighting the strengths and limitations of relying on any single data source alone.

The fair level of agreement measured from the Cohen's kappa statistic indicates there may be substantial non-random differences in the data captured by diagnostic codes and PHA forms. The observed level of agreement for both the ‘recent use’ (78.5%) and ‘history of any use’ (71.8%) classifications demonstrate some consistency between the 2 sources, although not reliably enough to substitute one for the other. They capture different populations, with the PHA more effective for younger service members and the Marine Corps, while diagnostic codes play an increasing role with advancing age. Therefore, a combined surveillance strategy may be critical for accurately monitoring current behaviors and informing targeted interventions.

The assignment of a diagnostic code for tobacco use is often illness-driven, meaning it is more likely to be recorded when a service member seeks care for a related health issue. Consequently, diagnostic codes may over-represent individuals already experiencing tobacco-related health problems, while the PHA provides a broader, more routine representation of use. This may explain why PHA-exclusive data skew heavily toward younger service members, particularly those under age 25 years, who may not yet have developed chronic conditions that would trigger a diagnostic code during a health care visit.

The Marine Corps also represented a higher service-specific proportion of PHA-exclusive data for both recent and historical use. This finding may be due to different health care-seeking behaviors in age groups and branches of service. As the PHA is completed with health care provider supervision, ^6^ self-reporting could be biased to avoid perceived negative repercussions of reporting, or could be skewed by service specific traditions related to tobacco use or health care seeking. ^10 , 11^

This study aggregated multiple diagnostic codes into a single category for tobacco and nicotine use. It is important, however, to recognize the distinctions between these codes, as their combination can make the results of comparative analyses difficult to interpret. Clinical practice uses specific ICD-10 codes to differentiate between active tobacco use (Z72.0), nicotine dependence (F17.2*), and personal history of nicotine dependence (Z87.891). While a health care provider can assign a tobacco use diagnosis with individual discretion, a diagnosis of tobacco dependence requires specific clinical and diagnostic criteria. This clinical nuance is not captured in the PHA's more generalized, self-reported, checked-box format, which focuses on current use patterns rather than formal diagnoses of dependence or historical use.

This assessment is subject to additional limitations. A PHA is recorded as overdue if not completed by 90 days after the due date; thus, ACSMs who were overdue for an annual PHA may not be represented. Additionally, the evaluation of tobacco and nicotine use from medical records may be affected by incomplete data due to coding practices. Although medical codes are specifically allocated to indicate smoking status, an important challenge is whether providers properly document this behavioral risk factor in administrative claims data, which are generated for billing purposes. ^14^ Additionally, incomplete data may pose a limitation for occurrences of missing medical records. The issue of missing medical records for this cohort is not a significant concern, however. Of the 921,394 ACSMs who completed a PHA in 2023, only 328 had no medical records over the preceding 20-year period.

This analysis did not query open text fields for non-structured data documented on the PHA or within medical record chart notes. One analysis found that 35% of individuals with no structured ICD code for ‘former smoker’ had this information in some form in open text notes on their chart. ^9^ Free-text analysis of the standardized PHA survey can provide valuable insights into emerging tobacco and nicotine products not captured by checked-box responses. For instance, nicotine pouches—dissolvable microfiber pouches of nicotine salt powder—have recently gained popularity in the U.S. ^15^ A convenience-based, self-reported survey indicated that nicotine pouch use among U.S. military personnel is 10 times higher than in the general adult population. ^16^ Although the current study did not specifically measure nicotine pouch use, a total of 9,630 ACSMs completing a PHA in 2023 reported using an “other” tobacco product. Analyzing the free-text responses associated with this ‘other’ category may reveal more information about emerging product use.

These data are meant to inform future case-finding development processes for tobacco and nicotine use. Additional modifications to the surveillance definitions or matching processes may be required, either to improve estimation accuracy or simplification for routine processing. The 30-day overlap period provided a crude method to match PHA responses with medical records, to account for any variance in 30-day reporting and potential delays in diagnostic documentation. Thus, additional consideration may be required to improve precision of timing between PHA responses and medical diagnostic codes.

The results of this study indicate that neither self-reported PHA data nor medical diagnostic codes alone provide a complete picture of tobacco and nicotine use among ACSMs. The fair level of agreement between sources, particularly for recent use, highlights that each method captures a demographically distinct subset of users, underscoring the need for a multi-faceted approach to public health surveillance. This combined methodological approach may enhance future public health surveillance, improve the accuracy of epidemiological studies, and ultimately provide a stronger evidence base for policies aimed at improving the health and readiness of the force.

## References

[1] Public Health Service, Office of the Surgeon General ; Samet JM , Pechacek TF , Norman LA , Taylor PL , eds. The Health Consequences of Smoking—50 Years of Progress: A Report of the Surgeon General . Appendix 14.1: tobacco control efforts in the Department of Defense . U.S. Dept. of Health and Human Services ; 2014 . Accessed Dec. 1, 2025 . https://www.hhs.gov/sites/default/files/consequences-smoking-appendix14-1-tobacco-control-efforts.pdf

[2] Public Health Service, Office of the Surgeon General ; Fagan F , Hickman NJ , Kennedy R , et al , eds. Eliminating Tobacco-Related Disease and Death: Addressing Disparities—A Report of the Surgeon General . U.S. Dept. of Health and Human Services ; 2024 . Accessed Dec. 1, 2025 . https://www.hhs.gov/sites/default/files/2024-sgr-tobacco-related-health-disparities-full-report.pdf 40373173

[3] Patrick S , Boyle C , LaMorte D , Dore M. Tobacco use prevalence in the Military Health System: a retrospective study . Mil Med . 2024 ; 189 ( 11-12 ): e2632 - e2637 . doi: 10.1093/milmed/usae208 38743582

[4] Institute of Medicine Committee on Smoking Cessation in Military and Veteran Populations ; Bondurant S , Wedge R , eds. Combating Tobacco Use in Military and Veteran Populations . National Academies Press ; 2009 . Accessed Mar. 4, 2026 . doi: 10.17226/12632 25009944

[5] Meadows SO , Engel CC , Collins RL , et al . 2018 Department of Defense Health Related Behaviors Survey (HRBS): Results for the Active Component . RAND Corp .; 2021 . Accessed Dec. 1, 2025 . https://www.rand.org/pubs/research_reports/RR4222.html 10.7249/rhq-08-02-05 PMC6183770 30323988

[6] Office of the Under Secretary of Defense for Personnel and Readiness . DOD Instruction 6200.06. Periodic Health Assessment (PHA) Program . U.S. Dept. of War . Updated Apr. 18, 2025 . Accessed Dec. 1, 2025 . https://www.esd.whs.mil/Portals/54/Documents/DD/issuances/dodi/620006p.pdf

[7] Mancuso JD , Ahmed AE , Rossi KR. Tobacco and nicotine use among active component U.S. service members: a comparison of the 2018 estimates from the Health Related Behaviors Survey and the Periodic Health Assessment . MSMR . 2024 ; 31 ( 3 ): 2 - 12 . Accessed Dec. 1, 2025 . https://www.health.mil/news/articles/2024/03/01/msmr-tobacco-nicotine-use PMC11023702 38621256

[8] Nishi SPE , Zhou J , Young-Fang J , Sharma G , Goodwin J. Trends in tobacco use and tobacco cessation counseling codes among Medicare beneficiaries, 2001–2014 . BMC Health Serv Res . 2019 ; 19 ( 548 ): 1 - 9 . doi: 10.1186/s12913-019-4368-7 31382958 PMC6683517

[9] Ruckdeschel JC , Riley M , Parsatharathy S , et al . Unstructured data are superior to structured data for eliciting quantitative smoking history from the electronic health record . JCO Clin Cancer Inform . 2023 ; 7 ( 7 ). doi: 10.1200/cci.22.00155 36809022

[10] Nelson JP , Pederson LL , Lewis J. Tobacco use in the Army: illuminating patterns, practices, and options for treatment . Mil Med . 2009 ; 174 ( 2 ): 162 - 169 . doi: 10.7205/milmed-d-01-2008 19317197

[11] Britt TW , Sipos ML , Klinefelter Z , Adler A. Determinants of mental and physical health treatment-seeking among military personnel . Br J Psychiatry . 2020 ; 217 ( 2 ): 420 - 426 . doi: 10.1192/bjp.2019.155 31258095

[12] Armed Forces Health Surveillance Division . Surveillance Case Definition for Nonmedical Factors Influencing Health: Social, Environmental, and Behavioral . U.S. Dept. of War . Jul . 1 , 2024 . Accessed Mar. 17, 2026 . https://health.mil/reference-center/publications/2024/07/01/nonmedical-factors-influencing-health-social-environmental-behavioral

[13] Armed Forces Health Surveillance Division . Surveillance Case Definition for Clinical Over-weight . U.S. Dept. of War . Oct . 1 , 2016 . Accessed Mar. 17, 2026 . https://health.mil/reference-center/publications/2016/10/01/overweight-obesity

[14] Huo J , Yang M , Shih , Y. Sensitivity of claims-based algorithms to ascertain smoking status more than doubled with meaningful use . Value Health . 2018 ; 21 ( 3 ): 334 - 340 . doi: 10.1016/j.jval.2017.09.002 29566841

[15] Majmundar A , Okitondo C , Xue A , et al . Nicotine pouch sales trends in the US by volume and nicotine concentration levels from 2019 to 2022 . Subst Use Addctn . 2022 ; 5 ( 11 ): e2242235 . doi: 10.1001/jamanetworkopen.2022.42235 PMC9667333 36378312

[16] Little MA , Polaskey KM , Pilehvari A , Krukowski RA , Ribisl KM. Nicotine pouch use among US military personnel . JAMA Netw Open . 2024 ; 7 ( 12 ): ee2451517 . doi: 10.1001/jamanet-workopen.2024.51517 PMC11653119 39688869

